# Glial fibrillary acidic protein autoimmunity in reversible splenial lesion syndrome: diagnostic and therapeutic implications

**DOI:** 10.3389/fneur.2025.1650256

**Published:** 2025-09-29

**Authors:** Lingling Lin, Xiang Li, Chao Quan, Jingzi Zhangbao, Hongmei Tan, Yi Wang, Siyuan Xu, Zhihao Dai

**Affiliations:** ^1^Department of Neurology, The Third Affiliated Hospital of Wenzhou Medical University, Wenzhou, Zhejiang, China; ^2^Department of Neurology, Huashan Hospital, Fudan University, Shanghai, China; ^3^The Second Clinical Medical College, Zhejiang Chinese Medical University, Hangzhou, China

**Keywords:** autoimmune glial fibrillary acidic protein astrocytopathy (GFAP-A), GFAP, splenium of corpus callosum, corpus callosum, RESLES, viral encephalitis

## Abstract

Autoimmune GFAP astrocytopathy (GFAP-A) is a neuroinflammatory condition that often involves the brain, meninges, and spinal cord. Its characteristic MRI finding consists of linear or radial perivascular enhancement adjacent to the ventricles. While corpus callosum splenium lesions occur in only 5% of cases, association with reversible splenial lesion syndrome (RESLES) is very rare. In such instances, GFAP-A can clinically resemble viral encephalitis, making diagnosis difficult. This article discusses how to distinguish GFAP-A from viral encephalitis using clinical and auxiliary examinations when RESLES is present.

## 1 Introduction

In 1999, Kim et al. (1) first reported a reversible, ovoid, non-hemorrhagic splenial lesion on MRI, which is often linked to AED toxicity and demyelination. Since then, advances in neuroimaging and clinical research have significantly expanded the understanding of splenium of the corpus callosum (SCC)lesions. Garcia-Monco et al. (2) introduced the term Reversible Splenial Lesion Syndrome (RESLES) in 2011 for a distinct clinicoradiological entity with a diversity of etiologies. This entity is defined by transient, oval-shaped, non-enhancing lesions in the splenium of the corpus callosum (SCC) on MRI that typically resolve spontaneously. Since then, there have been numerous case reports of RESLES. There are diverse etiologies that can cause RESLES, including seizures, withdrawal of antiepileptic drugs, effects of other medications, infections, metabolic disorders, intoxication, malignant tumors, among others (3).

Glial fibrillary acidic protein (GFAP), an intermediate filament protein predominantly expressed in mature astrocytes, can become a target of autoimmune attack, resulting in autoimmune GFAP astrocytopathy (GFAP-A). The disease typically presents with acute or subacute onset, frequently preceded by prodromal symptoms such as headache, fever, or other flu-like manifestations. Over subsequent days to weeks, the clinical course may progress to include a broader spectrum of neurological deficits, including movement disorders, visual disturbances, psychiatric symptoms, and autonomic dysfunction (4). On neuroimaging, GFAP-A typically demonstrates linear periventricular radial enhancement on post-contrast T1-weighted MRI, observed in approximately 53% of cases (5). Corpus callosum involvement in GFAP-A is uncommon, occurring in ~5% of cases (6–9), and frequently coexists with RESLES.

Approximately 29% of GFAP-A patients report influenza-like prodromal symptoms prior to neurological onset of neurological manifestations (10). In clinical practice, distinguishing GFAP-A from viral encephalitis can be challenging—particularly when anti-GFAP antibody test results are pending or when testing is not performed. Misdiagnosis is not uncommon, with several documented cases initially attributed to viral encephalitis (9, 11, 12). This article focuses on differentiating GFAP-A from viral encephalitis in patients with RESLES. Early and accurate discrimination between these two entities is essential to guide appropriate immunotherapy, prevent treatment delays, and ultimately improve patient outcomes.

## 2 Materials and methods

### 2.1 Selection of study participants

#### 2.1.1 GFAP-A cohort

This retrospective study included patients diagnosed with GFAP-A at Huashan Hospital of Fudan University. Inclusion criteria comprised (1) positive GFAP-IgG in cerebrospinal fluid (CSF) and (2) imaging findings consistent with reversible splenial lesion syndrome (RESLES), with exclusion of alternative diagnoses. Four eligible cases were identified. Comprehensive medical records—encompassing demographic characteristics, clinical presentation, laboratory results, and neuroimaging data—were systematically reviewed to verify diagnoses and ensure eligibility.

A systematic literature review was performed in PubMed for articles published (4, 7, 9, 11–15) using the search terms: “GFAP-A,” “RESLES,” and “autoimmune glial fibrillary acidic protein astrocytopathy.” All identified publications reporting GFAP-A cases with concurrent RESLES were evaluated for inclusion (Table 1).

**Table 1:** GFAP-A cohort.

**Author /case**	**Clinical manifestations**	**Pathogens**	**HypoNa**	**CSF features**				MRI^*^	GFAP-Ig G^**^	**Treatment**	Prognosis^***^
				**CC**	**Prot**.	**Glu**	**Cl**				
Case 1	Fever, headache, mental abnormality, hallucination, somnolence, weakness in both lower extremities, urinary retention, intestinal obstruction tremor	CSF NGS (-)	Exist	↑	↑	↓	↓	Leptomeningeal enhancement (cervical spine, thoracic spine, conus)	1:32/(-)	Corticosteroid therapy, Efgartigimod Alfa Injection, IVIG	5
Case 2^****^	Fever, mental abnormality, tremor, somnolence, intestinal obstruction, epilepsy	CSF NGS suspiciously detected EBV-DNA	Exist	↑	↑	↓	↓	Periventricular linear enhancement and cervical/thoracic leptomeningeal enhancement	1:100/1:32	Corticosteroid therapy, IVIG	2
Case 3	Fever, headache, vomiting, urinary retention	CSF NGS (-)	Exist	↑	↑	↓	↓	No lesions	1:3.2/1:10	Corticosteroid therapy, IVIG	1
Case 4	Fever, headache	CSF NGS (-)	Exist	↑	↑	↓	↓	Cranial pial linear enhancement with cervical/thoracic leptomeningeal enhancement	1:10/ ND	Corticosteroid therapy, IVIG	0
Wang et al. (9)	Fever, both temporal pain, low limbs fatigue, frequent urination	CSF NGS (-)	Exist	↑	↑	↓	↓	No lesions	1:32/(-)	Corticosteroid therapy	0
Lin et al. (7)	Fever, headache, confusion, weakness in both lower limb, acute delirium, urinary retention, epilepsy, limited bilateral abduction	CSF NGS (-)	ND	↑	↑	↓	ND	Abnormal leptomeningeal enhancement in the brainstem	1:10/1:10	Corticosteroid therapy, (mycophenolate mofetil)	1
Oger et al. (13)	Fever, vomiting, headache, alteration of consciousness„ dysmetria, nystagmus, gait difficulties	CSF PCR (HSV1/2, VZV, EV, CMV, EBV, HHV-6)(-)	Exist	↑	↑	ND	ND	No lesions	(+)/ND	IVIG	0
Nakamura et al. (4)	Fever, headache, urinary retention, constipation, myoclonus of the upper limbs	CSF PCR (tuberculosis)(-)	Exist	↑	↑	↓	ND	Transient appearance of hyper-intensity in the bilateral putamen	(+)/ND	Corticosteroid therapy	0
Issa et al. (11)	Fever, headache, hallucinations, confusion, tetraplegia, acute respiratory failure	NGS:HPIV-3	ND	↑	↑	ND	ND	No lesions	(+)/ND	IVIG, Glucocorticoid therapy, PE, RTX, CTX	2
Guo et al. (14)	Headache, psychosis, weakness in both lower extremities, coma, seizures, blurred vision, hypoventilation	CSF PCR(-) NGS(-)	ND	↑	↑	ND	ND	No lesions	1:10/(-)	Corticosteroid therapy, IVIG	0
Hréaud et al. (12)	Fever, headaches, diplopia, walking difficulties, ataxia of the lower limbs, altered consciousness, left facial paralysis, urinary retention, dysarthria, complete visual loss in the left eye	PCR (HSV-1/2, VZV, CMV, tuberculosis, syphilis, Lyme, or intracellular bacteria)	Exist	↑	↑	↓	ND	Diffuse leptomeningeal gadolinium enhancement in the cervical spinal cord, leptomeningeal gadolinium enhancement of the optic nerves	(+)/ND	Corticosteroid therapy	0
Ahmed et al. (15)	Fever, headache, altered sensorium, urinary retention, diplopia, gait ataxia, bilateral papilledema	Viral encephalitis panel(-)	Exist	↑	↑	Normal	ND	Conus leptomeningeal enhancement	(+)/(+)	Methylprednisolone	0

^*^MRI Features: excluding corpus callosum lesions; ^**^ GFAP antibody: cerebrospinal fluid/serum.

^***^Outcome: Modified Rankin Scale (mRS); ^****^Case 2 positive for NMDAR antibodies, Serum NMDAR antibody: 1:32, CSF NMDAR antibody: 10.

HypoNa, hyponatremia; CC, Cell Counts; Prot., Protein; Glu, Glucose; Cl, Chloride; CSF, cerebrospinal fluid; NGS, Next-Generation Sequencing; PCR, polymerase chain reaction; HSV-1, Herpes Simplex Virus Type 1; HSV-2, Herpes Simplex Virus Type 2; EV, Enterovirus; VZV, Varicella-Zoster Virus; CMV, Cytomegalovirus; EBV, Epstein-Barr Virus; HHV-6, Human Herpesvirus Type 6; HPIV, human parainfluenza virus; RTX, Rituximab; CTX, Cyclophosphamide; IVIG, Intravenous Immunoglobulin; ND, not described in detail.

Mental abnormality: delirium, rave.

↑, Higher than the normal value; ↓, Lower than the normal value.

#### 2.1.2 Viral encephalitis cohort

For the viral encephalitis cohort, a total of 10 cases were collected, including one retrospectively enrolled inpatient case from Huashan Hospital of Fudan University and nine cases identified through PubMed database search of viral encephalitis with concomitant RESLES (16–23). Inclusion criteria for viral encephalitis: supported by etiological evidence, presenting with neurological symptoms of encephalitis, exclusion of other diseases, and imaging findings consistent with RESLES (Table 2). The cases of viral encephalitis had clear evidence of the pathogen and other diseases were excluded.

**Table 2:** Viral encephalitis cohort.

**Author**	**Clinical manifestations**	**Pathogen**	**Hyponatremia**	**CSF Features**	MRI features^*^	**Treatment**	**Prognosis**
Case 5	Fever, incoherent speech	SARS-CoV-2	NO	Normal	No lesions	Ganciclovir Injection	0
Takanashi et al. (20) Case	Fever, cough, rhinorrhea, disorientation, hallucinations	Influenza A (H3)	NO	Normal	No lesions	Amantadine	0
Takanashi et al. (20) Case	Fever, cough, rhinorrhea, right-side-dominant paralysis, facial palsy	Influenza B	NO	Normal	No lesions	Oseltamivir phosphate	0
Kimura et al. (19)	Fever, mild painful throat, myalgia, arthralgia, tetraplegia, transcortical motor aphasia, mildly altered mental status	Influenza type A virus	NO	Normal	No lesions	Methylprednisolone	0
Takatsu et al. (16)	Fever, disordered consciousness, decreased olfaction	Influenza A	NO	Normal	No lesions	No treatment	0
Balagopal et al. (23)	Fever, vesicular skin rash, epilepsy, altered sensorium	Varicella zoster	NO	Lymphocytic pleocytosis with raised protein	No lesions	Acyclovir, anticonvulsants, steroids	0
Fu et al. (22)	Fever, headache, vomiting	Cytomegalovirus	Exist	elevated protein	No lesions	Ganciclovir	0
Nagae et al. (17)	Fever, altered mental status	COVID-19	NO	Normal	No lesions	Remdesivir	0
Matsubara et al. (18)	Fever, headache, severely lethargic, exhibited muscle weakness of the extremities	Influenza B	NO	Normal	No lesions	Methylprednisolone	0
Hagemann et al. (21)	Fever, sickness, dizziness, ataxia, nystagmus, confusion, epilepsy	Epstein-Barr Virus	NO	Lymphocytic pleocytosis with raised protein	MRI T2 FLAIR, DWI signal intensity hyperintensities in Parieto-occipital, cortical areas	Acyclovir, ceftriaxone, ampicillin, High-dose steroids, anticonvulsive treatment	0

^*^MRI features: excluding corpus callosum lesions. MRI, Magnetic Resonance Imaging; FLAIR, Fluid-Attenuated Inversion Recovery; DWI, Diffusion Weighted Imaging.

### 2.2 Methods

For both cohorts, we systematically extracted and analyzed data encompassing clinical presentations, laboratory results, treatment regimens, and clinical outcomes. All statistical analyses were conducted using SPSS software (version 29.0; IBM Corp). Categorical variables were compared using Fisher's exact test, with a two-tailed *P*-value < 0.05 considered statistically significant.

The study protocol adhered to the ethical guidelines of the Declaration of Helsinki. For the retrospectively collected cases from our institution, all patient data were de-identified prior to analysis, and the study was approved by the Institutional Review Board of Huashan Hospital of Fudan University. Cases obtained from published literature involved only secondary analysis of anonymized, publicly available data, thus maintaining strict patient confidentiality throughout the research process.

## 3 Results

GFAP-A group (*n* = 12): anti-GFAP antibodies were detected positively in the cerebrospinal fluid of all cases. Additionally, Case 2 tested positive for anti-N-methyl-D-aspartate receptor (NMDAR) antibodies in both serum and cerebrospinal fluid. Universal CSF findings included pleocytosis and elevated protein, with post-treatment improvement in 6 of 12 cases (6 lacked follow-up) and hypoglycorrhachia in 8 cases. Refractory hyponatremia was observed in 9 cases despite sodium supplementation (Table 1).

**Figure 1:**
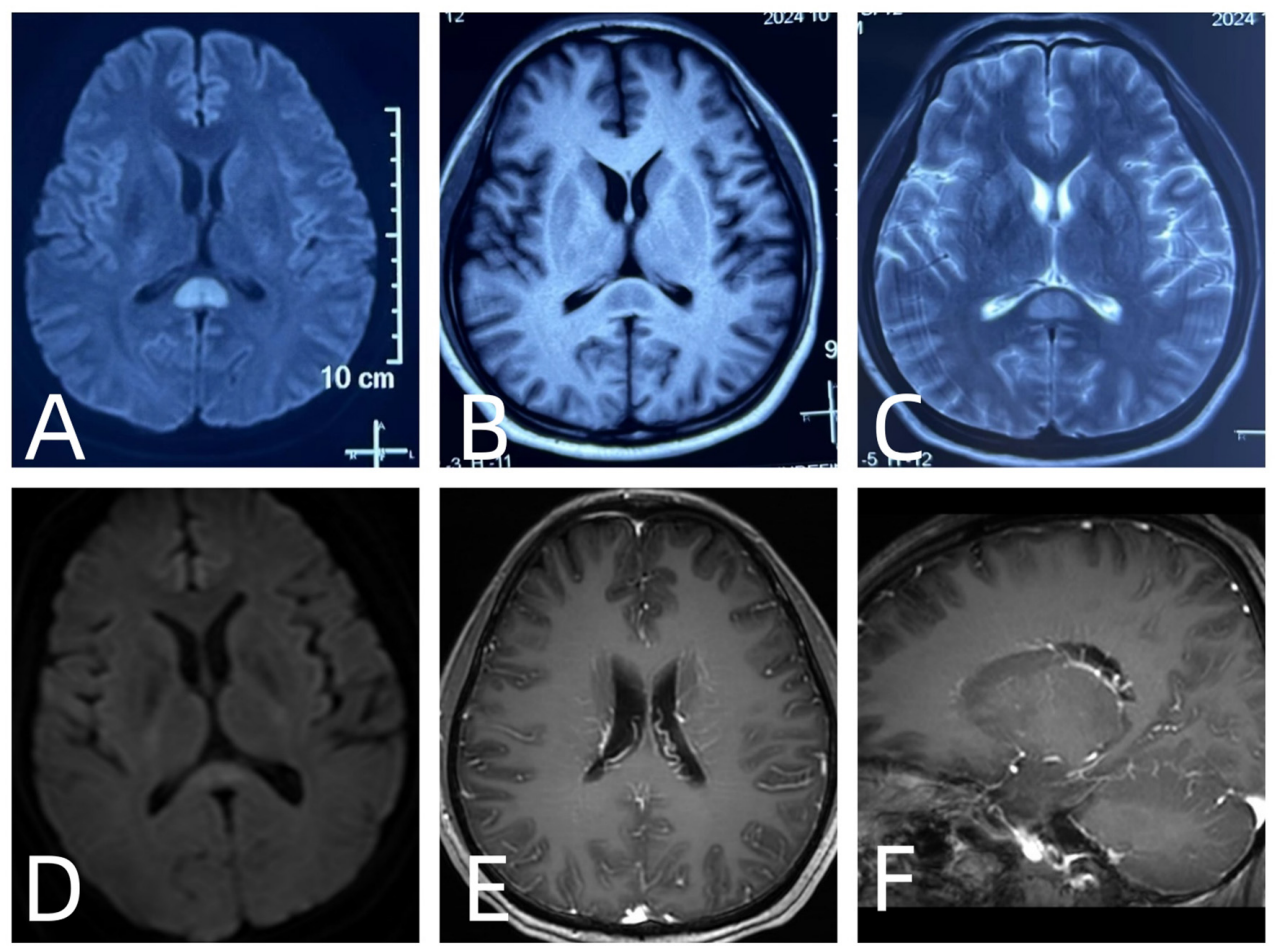
(**A)** diffusion-weighted imaging (DWI): hyperintense signal in the splenium of the corpus callosum. **(B)** T1-weighted imaging (T1WI): hypointense signal in the splenium of the corpus callosum. **(C)** T2-weighted imaging (T2WI): hyperintense signal in the splenium of the corpus callosum. **(D)** Follow-up DWI: resolution of the previously observed hyperintense signal in the splenium of the corpus callosum. **(E, F)** Post-contrast imaging: linear perivascular enhancement radiating outward from the lateral ventricles.

**Figure 2:**
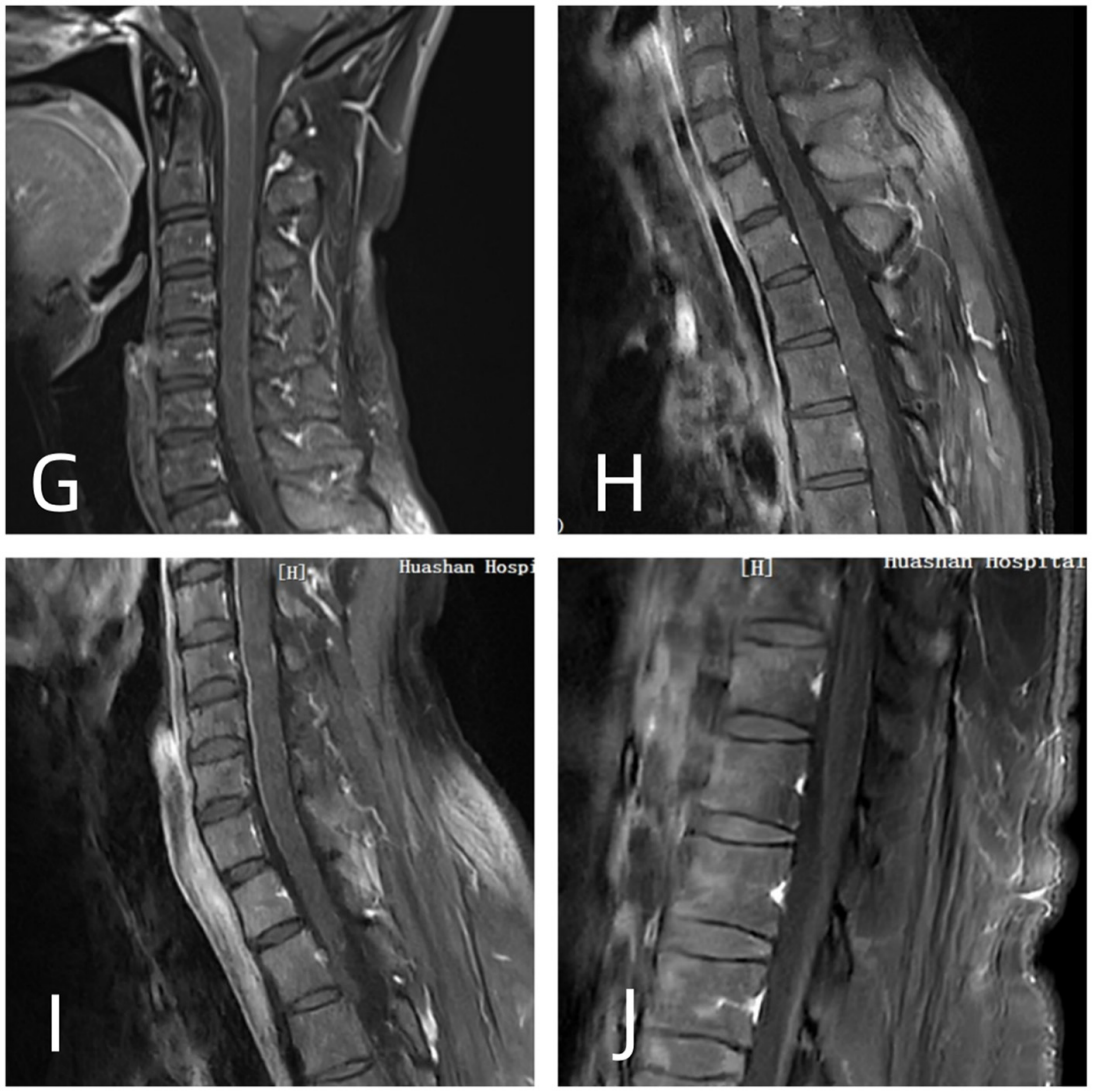
**(G, I)** T1-weighted contrast-enhanced (CE-T1WI): linear enhancement of the cervical spinal cord pia mater. **(H)** CE-T1WI: linear enhancement of the pial membrane in the thoracic spinal cord **(J)** CE-T1WI: leptomeningeal enhancement around the conus.

Brain MRI revealed RESLES-compatible abnormal signals in the SCC in all cases (Figure 1). Enhanced spinal MRI showed leptomeningeal enhancement in four cases (Figure 2). All patients received immunotherapy (corticosteroids, IVIG, or monoclonal antibodies), resulting in complete recovery in seven cases and neurological sequelae in five, with a maximum mRS score of 5.

In the viral encephalitis cohort, all cases were virologically confirmed and demonstrated characteristic RESLES findings on brain MRI. Cerebrospinal fluid (CSF) analysis showed an elevated cell count in two cases and increased protein levels in three cases, while glucose levels were within normal limits in all patients. Treatment regimens included antiviral therapy (7 cases), corticosteroid immunotherapy (4 cases), or no intervention (1 case). All patients recovered completely without neurological sequelae.

Table 3 presents a comparison between the GFAP-A and viral encephalitis cohorts in terms of gender, clinical manifestations, laboratory findings, treatment, and outcomes. No significant differences were observed in gender distribution, movement disturbances, or psychiatric symptoms. In contrast, urinary dysfunction was significantly more frequent in the GFAP-A group (6 vs. 0 cases; *p* = 0.015). Marked intergroup differences were noted in laboratory results: the GFAP-A cohort exhibited significantly higher rates of hyponatremia (9 vs. 1; *p* = 0.004), CSF pleocytosis (12 vs. 2; *p* < 0.001), hyperproteinorrhachia (12 vs. 3; *p* < 0.001), and hypoglycorrhachia (8 vs. 0; *p* = 0.002).

**Table 3:** Comparison between GFAP-A and viral encephalitis.

**Characteristics**	**GFAP-A**	**Viral encephalitis**	***P***
Number of cases	12	10	
Gender	Male 10, Female 2	Male 7, Female 3	0.624
Psychosis	5 (41.7%)	3 (30%)	0.675
Urinary dysfunction	6 (50%)	0	0.015
Movement Disturbances	10 (83.3%)	4 (40%)	0.074
Hyponatremia	9 (75%)	1 (10%)	0.004
Increased cell count in CSF	12 (100%)	2 (20%)	< 0.001
Increased protein levels in CSF	12 (100%)	3 (30%)	< 0.001
Low glucose levels in CSF	8 (66.7%)	0	0.002
Abnormalities in the spinal cord MRI	5 (41.6%)	0	0.04
Immunotherapy	12 (100%)	4 (40%)	0.003
Sequelae	5 (42.7%)	0	0.04

Psychosis: mental abnormality, confusion, hallucinations, incoherent speech.

Abnormal urination: urinary retention, frequent urination.

Movement disturbances:gait disturbance, ataxia, tremor, limb weakness, and myoclonus.

Psychosis: incoherent speech, mental abnormality (delirium, rave), hallucinations.

Not described in detail is considered normal.

Treatment strategies also differed markedly between the two groups, particularly regarding the use of immunotherapy. Clinical outcomes diverged significantly: five patients in the GFAP-A group developed neurological sequelae—primarily persistent urinary dysfunction, including one severe case with an mRS score of 5—while all viral encephalitis patients achieved complete recovery without residual deficits (*p* = 0.035).

In summary, GFAP-A and viral encephalitis demonstrated significant clinical differences across multiple domains: (1) clinical manifestations—with distinct prevalence of urinary dysfunction in GFAP-A; (2) laboratory parameters—showing marked differences in hyponatremia incidence and CSF profiles (pleocytosis, protein elevation, and hypoglycorrhachia); and (3) prognostic outcomes—with neurological sequelae exclusively observed in GFAP-A cases. These divergent features form evidence-based diagnostic parameters for accurate clinical differentiation.

## 4 Discussion

GFAP-A is a rare neuroinflammatory disorder. Its most common clinical manifestations include meningoencephalomyelitis (32%), meningoencephalitis (24%), and encephalitis (12%) (24). These presentations closely resemble those of viral encephalitis, which typically presents with acute onset, fever, altered consciousness (ranging from confusion to coma), and seizures (both focal and generalized) (25). Notably, several viruses—including influenza, rotavirus, measles, adenovirus, human parvovirus B19, cytomegalovirus, and EB virus—are known to cause encephalitis with characteristic splenial lesions of the corpus callosum (26). The clinical similarity between GFAP-A and viral encephalitis poses significant diagnostic challenges. However, CSF analysis provides key differentiating features: while viral encephalitis typically shows lymphocytic pleocytosis with normal glucose and normal/mildly elevated protein levels (25), GFAP-A often demonstrates more pronounced CSF abnormalities (5), including marked protein elevation and, in some cases, increased adenosine deaminase (ADA) levels (27) -findings uncommon in viral encephalitis.

Retrospective studies indicate that 54%−84% of GFAP-A patients exhibit cerebrospinal fluid (CSF) abnormalities, including pleocytosis (elevated white blood cell count) and increased protein levels (28). Notably, CSF pleocytosis is considered a key diagnostic biomarker for GFAP-A (10), a finding consistent with our analysis. However, the clinical significance of CSF abnormalities—including their correlation with disease severity and long-term prognosis—remains unclear and warrants further investigation.

In the GFAP-A cohort, decreased CSF glucose levels were observed in 8 cases (66.7%), a finding that showed a statistically significant difference when compared to the control group. A similar phenomenon was reported in 18% of the 102 patients included in the Mayo Clinic cohort (10), although the underlying mechanism remains unclear and requires further investigation.

Although no statistically significant difference in movement disturbances was observed between the GFAP-A and viral encephalitis groups in our cohort, literature reports indicate (24) that 59% of GFAP-A patients develop motor dysfunction—manifested as gait disturbances, ataxia, tremors, limb weakness, or myoclonus—while 38% exhibit autonomic dysfunction. Urinary dysfunction and hyponatremia are key indicators in identifying suspected cases of GFAP-A (27), a pattern that aligns with our observational findings. In clinical practice, splenial lesions of the corpus callosum on neuroimaging often raise initial suspicion for viral causes. However, when such radiological features co-occur with motor or autonomic dysfunction, GFAP-A should be considered an important differential diagnosis. We therefore recommend prompt testing for GFAP-IgG antibodies in these scenarios and initiating immunotherapy promptly upon confirmation.

The above analysis suggests that RESLES patients can be preliminarily differentiated between GFAP-A and viral encephalitis through distinct clinical and laboratory features. Clinically, the co-occurrence of motor dysfunction and urinary dysfunction strongly favors GFAP-A. Laboratory findings including hyponatremia, CSF pleocytosis (typically with normal glucose but elevated protein levels) provide additional discriminative value in distinguishing these two conditions.

## 5 Conclusion

RESLES is a clinically and etiologically heterogeneous syndrome. While most patients have a favorable prognosis with appropriate treatment, accurate etiological diagnosis is essential. In clinically ambiguous cases, comprehensive GFAP antibody testing in both serum and CSF is recommended, along with attention to characteristic features of GFAP-A such as autonomic dysfunction, hyponatremia, and CSF abnormalities (pleocytosis, elevated protein, or hypoglycorrhachia). Pending test results, early presumptive diagnosis may facilitate prompt initiation of corticosteroid therapy. Serial MRI follow-up is also advised to monitor radiological progression.

## 6 Limitation

Our study has several limitations. First, its retrospective nature may introduce potential biases in data collection and interpretation. Additionally, the small sample size limits the statistical power and generalizability of our findings, underscoring the need for further validation through larger, prospective studies. Moreover, in clinical practice, viral infections may potentially trigger GFAP-A, and viral encephalitis can exhibit features overlapping with those of GFAP-A, necessitating careful differential diagnosis by clinicians based on individual patient presentations.
